# Patterns and rates of abdominal lymphatic metastasis following esophageal carcinoma

**DOI:** 10.1371/journal.pone.0185424

**Published:** 2017-10-10

**Authors:** Junqiang Chen, Wenjie Cai, Yu Lin, Yuanmei Chen, Qingfeng Zheng, Jianji Pan, Chuanben Chen

**Affiliations:** 1 Department of Radiation Oncology, Fujian Cancer Hospital & Fujian Medical University Cancer Hospital, Fuzhou, China; 2 Departments of Radiation Oncology, First Hospital of Quanzhou Affiliated to Fujian Medical University, Quanzhou, Fujian, China; 3 Departments of Surgery, Fujian Cancer Hospital & Fujian Medical University Cancer Hospital, Fuzhou, China; University Hospital Llandough, UNITED KINGDOM

## Abstract

**Aim:**

To determine the rate of abdominal lymph node metastasis after radical surgery for esophageal cancer and define the radiotherapy target area.

**Methods:**

Of the 1593 patients who underwent R0 radical esophagectomy for thoracic esophageal squamous cell carcinoma (TE-SCC), 148 developed abdominal lymph node (LN) metastases within three years of surgery. During that time interval, patients were examined by various imaging methods (enhanced computer tomography, magnetic resonance imaging, and positron emission tomography–CT) at set time points. The emerging recurrence pattern, preferred sites for abdominal metastasis, and correlation with added clinical factors were carefully recorded, to permit for delineation of a target area for radiotherapy.

**Results:**

We found postoperative metastatic abdominal LNs in 9.3% of the patients treated for esophageal cancer. Lesions in the upper, middle, and lower esophageal segments metastasized to abdominal LNs at 2.3%, 7.8%, and 26.6% (P < 0.0001), respectively. Of all cases, 4.8% had fewer than two affected LNs, while 20.1% had more than three metastatic LNs (P< 0.0001). The metastasis rates of negative and positive celiac LNs were 4.6% and 22.7%, respectively. Abdominal LN metastasis rates for the following LNs: 16a2 and 16a1 of para-aortic, celiac artery, posterior surface of the pancreatic head and common hepatic artery were 64.9%, 41.2%, 37.8%, 32.4%, and 20.9%, respectively. The overall rate of metastasis to these groups of LNs was 91.9%.

**Conclusion:**

This study determined that stations 16a1 and 16a2 of the para-aortic, truncus coeliacus, posterior surface of the pancreatic head, and arteria hepatica communis lymph nodes were the preferred sites for abdominal LN metastasis, thus defining target areas for postoperative radiotherapy.

## Introduction

Local metastasis to abdominal lymph nodes (LNs) is the main cause for failure to respond to radical surgery in patients with esophageal cancers, mostly due to the high recurrence frequency (8.4%–20%) [1–5]. Previous studies [1,6] reported that postoperative radiotherapy had a positive impact on patient survival after radical surgeries for stage III and LN-positive esophageal cancers, by reducing the rate of metastasized supraclavicular and upper mediastinal LNs. However, the overall rate of metastasis to LNs remained unchanged, since the target area for postoperative radiotherapy did not include the abdominal region [7]. Rates and patterns of postsurgery abdominal LN metastasis have been described in previous systematic studies [1, 8], but radiotherapy solutions have not been proposed. In this study, we examined 148 patients with abdominal LN metastasis after R0 radical surgery for thoracic esophageal squamous cell carcinoma (TE-SCC) and we analyzed their relapse patterns and sites, to provide references for designing an appropriate target area for postoperative radiotherapy.

## Materials and methods

### Ethics statement

Study participants voluntarily agreed to participate in the study and provided written informed consent prior to enrollment. The study was approved by the Ethics Committee of the Teaching Hospital of Fujian Medical University and Fujian Provincial Cancer Hospital.

### Recruitment of participants

Our study is a retrospective study. Of the 2510 thoracic esophageal squamous cell carcinoma patients, who underwent radical R0 surgery at the Fujian Provincial Tumor Hospital in China, between February 2005 and April 2013, we selected 1593 to participate in this study. Participants were instructed to return periodically to the observing hospital for follow-up evaluations. Specifically, participants were examined every 3 months in the first year, then every 6 months in second and third year, and annually thereafter until the completion of the study, in April 2015. Abdominal LN metastases were detected by regular abdominal enhanced CT, MRI, and PET-CT (in some cases). Only the corresponding and first authors had access to information that could identify individual participants during or after data collection.

### Inclusion criteria

a. No retroperitoneal LN or distant hematogenous metastases detected by enhanced computer tomography (CT), during the presurgical chest and abdomen examination.

b. More than 15 LNs dissected during the neck/chest/abdomen three-field or chest/abdomen two-field lymphadenectomy.

### Exclusion criteria

a. Less than15 dissected LNs or palliative excision.

b. Postoperative pathology report indicating areas characteristic of nonsquamous cell carcinoma.

c. Unreliable imaging information regarding specific metastasis sites.

d. Preoperative neoadjuvant radio- or chemoradiotherapy.

### Diagnostic criteria

Target areas for postoperative adjuvant radio- or chemoradiotherapy included the supraclavicular draining LNs, the upper mediastinal draining LNs, the anastomosis, and the original esophageal bed [6,9,10].

Metastatic abdominal LNs were considered those with a transverse diameter larger than 10 mm [11,12]. Categorization standards for esophageal cancers put forth by the 7th edition of the American Joint Committee on Cancer (AJCC) were ambiguous, leading to the decision to use the abdominal LNs classification standards for gastric carcinoma [13]: No. 8 (Hepatic arterial lymph nodes), No. 9 (celiac artery LNs), No. 10 (splenic hilar LNs, including those adjacent to the splenic artery and distal to the pancreatic tail; those adjacent to the roots of the short gastric arteries; and those along the left gastro-epiploic artery and proximal to its first gastric branch), No. 11 (proximal splenic artery LNs from its origin to halfway between its origin and the pancreatic tail end), No. 12 (hepato-duodenal ligament LNs), No. 13 (LNs on the posterior surface of the pancreatic head), No. 14 (LNs along the superior mesenteric vein), No. 16 (16b1 = para-aortic LNs in the diaphragmatic aortic hiatus; 16a1 = para-aortic LNs between the upper margin of the celiac artery origin and the lower border of the left renal vein; 16b1 = LNs adjacent to the abdominal aorta from the lower left renal vein to upper inferior mesentery artery; 16b1 = para-aortic LNs between the lower border of the left renal vein and the upper border of the inferior mesenteric artery origin), No. 18 (LNs along the inferior border of the pancreatic body), No. 19 (infra-diaphragmatic LNs predominantly along the subphrenic artery), and No. 20 (para-esophageal LNs in the diaphragmatic esophageal hiatus).

### Statistics

All data were analyzed using the SPSS15.0 software (SPSS Inc., Chicago, IL, USA). A chi-square test was used for statistical data comparison. The Kaplan–Meier method was adopted to calculate the survival rate, and the log-rank method was used to compare survival curves between groups. A Cox regression model with stepwise selection was used to perform multivariate analyses. *P*–values lower than 0.05 were considered statistically significant.

## Results

917 cases were excluded based on the following: 1)239 cases with esophageal cancer palliative resection or the number of surgical lymph node dissection was <15; 2) 136 cases with postoperative pathology report of non-squamous cell carcinoma; 3)170 cases of incomplete imaging data that prohibited accurate location of abdominal lymph node metastasis; 4) 372 cases with preoperative neoadjuvant chemotherapy or radiotherapy and chemotherapy.Following exclusion, 1593 patients were selected to participate in this study, with a median follow up duration of 43.5 months (95%CI: 38.4–48.6 months).

### Patterns of postoperative abdominal lymph node metastasis

148 presented with abdominal LN metastases at 1.1–74.4 months after surgery, with a median of 10.7 months. The abdominal metastasis rate post radical resection was 9.3%. Of all patients, 39.2% exhibited only abdominal LN metastases, 23.6% developed abdominal and other LN metastases, 19.6% had abdominal LN and hematogenous metastases, and 17.6% exhibited regional LN and hematogenous metastases. (Table 1).

**Table 1:** Pattern of postoperative abdominal lymph node metastasis (%, cases/all samples).

Metastasis pattern	Overall	Upper thoracic esophageal cancer	Middle thoracic esophageal cancer	Lower thoracic esophageal cancer
Abdominal lymph node metastasis only	39.2 (58/148)	0.0 (0/6)	41.4 (36/87)	40.0 (22/55)
Abdominal and other lymph node metastasis	23.6 (35/148)	50.0 (3/6)	19.5 (17/87)	27.3 (15/55)
Abdominal lymph node and hematogenous metastasis	19.6 (29/148)	16.7 (1/6)	19.5 (18/87)	18.2 (10/55)
Regional lymph node and hematogenous metastasis	17.6 (26/148)	33.3 (2/6)	18.4 (16/87)	14.5 (8/55)

### Rates of postoperative abdominal metastasis at specific lymphatic sites

**Fig 1:**
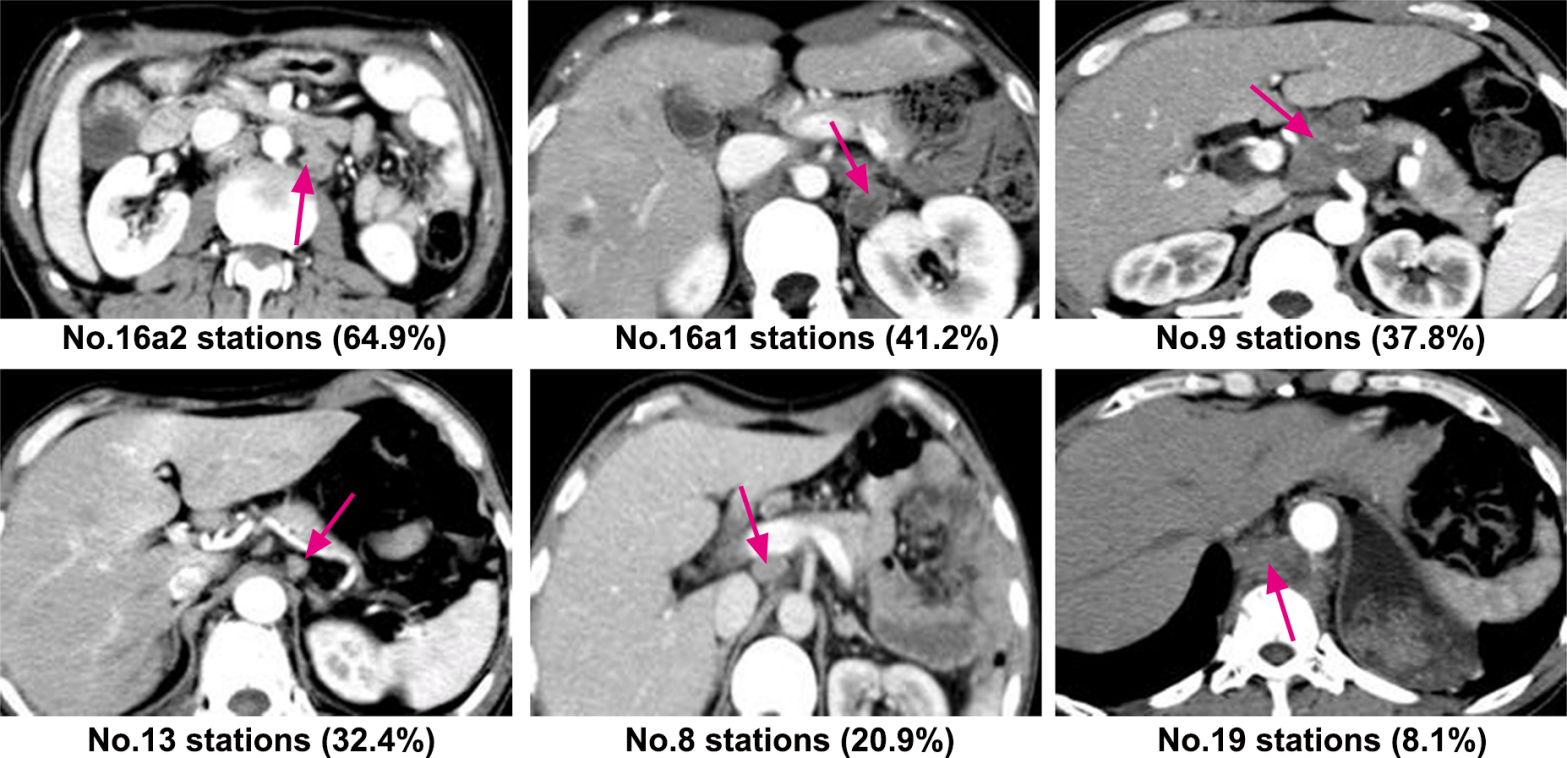
Specific sites with high rates of postoperative abdominal lymph node metastasis.

Of the 148 patients, seven had exceptionally high rates of postoperative abdominal LN metastasis, ranging from 6.8% to 64.9%, while the rest were below 5%. Abdominal metastasis rates at16a2 and 16a1 of the para-aortic, celiac artery, posterior surface of the pancreatic head, and common hepatic artery were 64.9%, 41.2%, 37.8%, 32.4%, and 20.9%, respectively. The overall metastasis rate in the above groups was 91.9%, as shown in Table 2.The illustration in Fig 1 shows specific sites with high rates of postoperative abdominal lymph node metastasis.

**Table 2:** Site distribution of postoperative abdominal LN metastasis in patients with TE-SSC (%, cases/all samples).

LN category	Overall	Upper thoracic esophageal cancer	Middle thoracic esophageal cancer	Lower thoracic esophageal cancer
No. 8	20.9 (31/148)	16.7 (1/6)	23.0 (20/87)	18.2 (10/55)
No. 9	37.8 (56/148)	33.3 (2/6)	36.8 (32/87)	40.0 (22/55)
No. 10	1.4 (2/148)	0.0 (0/6)	2.3 (2/87)	0.0 (0/55)
No. 11	4.1 (6/148)	0.0 (0/6)	5.7 (5/87)	1.8 (1/55)
No. 12	3.4 (5/148)	0.0 (0/6)	4.6 (4/87)	1.8 (1/55)
No. 13	32.4 (48/148)	33.3 (2/6)	27.8 (24/87)	40.0 (22/55)
No. 14	6.8 (10/148)	16.7 (1/6)	4.6 (4/87)	9.1 (5/55)
No. 15	1.4 (2/148)	0.0 (0/6)	1.1 (1/87)	1.8 (1/55)
No. 16	79.7 (118/148)	66.7 (4/6)	79.3 (69/87)	81.8 (45/55)
16a1	41.2 (61/148)	50.0 (3/6)	41.4 (36/87)	40.0 (22/55)
16a2	64.9 (96/148)	33.3 (2/6)	65.5 (57/87)	67.3 (37/55)
16b1	6.8 (10/148)	0.0 (0/6)	10.3 (9/87)	1.8 (1/55)
16b2	0.7 (1/148)	0.0 (0/6)	0.0 (0/87)	1.8 (1/55)
No. 18	2.7 (4/148)	0.0 (0/6)	3.4 (3/87)	1.8 (1/55)
No. 19	8.1 (12/148)	16.7 (1/6)	5.7 (5/87)	10.9 (6/55)
No. 20	1.4 (2/148)	0.0 (0/6)	1.1 (1/87)	1.8 (1/55)

### Correlation between clinical factors and postoperative rates of abdominal LN metastasis

The rates of abdominal metastasis from upper, middle, and lower esophageal thoracic cancers were 2.3%, 7.8%, and 26.6% (*P*< 0.0001), respectively. In patients with postoperative pathology of T1/2, the rate of abdominal metastasis was 8.7% and in those with T3/4, it was 9.5% (*P*< 0.0001). The rate of abdominal metastatic LNs in cases with other metastatic LNs was13.7% and in those without other metastases, it was 3.7% (*P*< 0.0001). 4.8% of cases had fewer than two abdominal lymphatic metastases and 20.1% had more than three (*P*< 0.0001). 4.6% of cases were negative for metastatic celiac LNs, while 22.7% were found to be positive. Abdominal metastasis in patients with and without postoperative adjuvant radio- or chemoradiotherapy occured at rates of 18.2% and 16.0%, respectively (*P* = 0.478) (Table 3). We analyzed the above six clinical factors and post-abdominal lymph node metastasis by multivariate regression analysis. The results indicated that lesion site, celiac lymph node metastasis, number of metastasic lymph nodes independently predict abdominal metastases (Table 4).

**Table 3:** Relevance of confounding clinical factors for the incidence of abdominal lymph node metastasis in TE-SCC patients (%, cases/all samples).

Factor	All patients	With abdominal lymph node metastasis	*χ*^2^ value	*P* -value
Sample size	1593	148		
Lesion site			92.041	< 0.0001
Upper segment of chest	16.7 (266/1593)	2.3 (6/266)		
Middle segment of chest	70.3 (1120/1593)	7.8 (87/1120)		
Lower segment of chest	13.0 (207/1593)	26.6 (55/207)		
Postoperative T classification			0.274	0.601
T1/2	30.9 (493/1593)	8.7 (43/493)		
T3/4	69.1 (1100/1593)	9.5 (105/1100)		
Presence of lymph node metastasis			46.900	< 0.0001
No	44.2 (704/1593)	3.7 (26/704)		
Yes	55.8 (889/1593)	13.7 (122/889)		
Number of metastasis lymph node			91.631	< 0.0001
0–2	70.6 (1125/1593)	4.8 (54/1125)		
≥ 3	29.4 (468/1593)	20.1 (94/468)		
Celiac lymph nodes			118.859	< 0.0001
Negative	73.9 (1178/1593)	4.6 (54/1178)		
Positive	26.1 (415/1593)	22.7 (94/415)		
Adjuvant therapy			0.504	0.478
No	83.8 (1335/1593)	9.1 (121/1335)		
Yes	16.2 (258/1593)	10.5 (27/258)		

**Table 4:** Multivariate regression analysis of clinical factors and abdominal lymph node metastasis in TE-SCC.

Factor	Chi-Square	P-value
Lesion site	39.458	0.000
Celiac lymph node metastasis	9.650	0.002
Number of metastasis lymph node	17.433	0.000
Postoperative T classification	2.179	0.140
Adjuvanttherapy	27.546	0.100
Presence of lymph node metastasis	0.015	0.901

## Discussion

Metastasis to lymph nodes is commonly complicating the outcome of esophageal cancers [11] and abdominal metastases, in particular, are responsible for failure of complete remission after radical surgery. Xiao *et al*.[1] reported an overall rate of metastasis of 8.4% after TE-SCC (41/486), while Liu *et al*.[8] reported a 14.8% rate (38/256). We determined an overall rate of 9.3% (148/1593), consistent with the reports from both publications [1,8].

According to previous studies [1–8,14], the metastasis rates vary greatly with the site of the cancerous lesion, ranging from 0%–8.3%, 8.1%–13.9%, and 26.8%–40.8% (*P*< 0.0001) for the upper, middle, and lower esophageal segments, respectively (Table 5). In this study, we also found the metastasis rates of the upper, middle, and lower esophageal segments to be higher with each segment, indicating increasing incidence for distally located lesions. One explanation for this observation could be the difference in the spreading mechanisms employed by cancers from different segments. Upper-segment esophageal cancers commonly metastasize to supraclavicular and upper mediastinal LNs and rarely to abdominal nodes. For middle-segment and lower-segment esophageal cancers, abdominal LN metastases were observed more frequently, while supraclavicular and upper mediastinal ones were quite rare[9].

**Table 5:** Rates of postoperative abdominal lymph node metastasis reported in other studies (%, cases/all samples).

Reference	Overall rate	Upper thoracic esophageal cancer	Middle thoracic esophageal cancer	Lower thoracic esophageal cancer
Cai WJ et al.^[^^3^^]^*	20.0 (28/140)	0.0 (0/7)	12.8 (11/86)	36.2 (17/47)
Doki Y et al.^[^^4^^]^*	16.7 (30/180)	3.6 (1/28)	8.1 (7/86)	33.3 (22/66)
Ge H et al.^[^^5^^]^*	14.5 (32/220)	5.2 (3/58)	15.7 (16/102)	23.3 (14/60)
Zhang WC et al.^[^^14^^]^*	20.0 (39/195)	8.3 (2/24)	13.9 (17/122)	40.8 (20/49)
Liu WJ et al.^[^^13^^]^ ^†^	14.8 (38/256)	3.1 (1/32)	12.1 (19/157)	26.8 (18/67)
Current study^†^	9.3 (148/1593)	2.3 (6/266)	7.8 (87/1120)	26.6 (55/207)

*Analysis for patients with lymph node metastasis after esophagectomy only.

^†^Analysis for the whole group with lymph node metastasis.

Our data showed that retroperitoneal LN metastasis was the major contributing factor for abdominal LN metastasis after radical surgery for esophageal cancers. The highest rates of metastasis were at stations 16a1 and 16a2 of the para-aortic, celiac artery, posterior surface of the pancreatic head, and common hepatic artery, ranging from highest to lowest. LNs in the vicinity of the cardia and arteria gastrica sinistra, which are frequently metastatic before the surgery, showed no signs of postoperative metastasis. This can be attributed to the complete removal of those LNs during surgery, as they are readily exposed and easy to access. Meanwhile LNs in the truncus coeliacus, on the posterior surface of the pancreatic head, and adjacent to arteria hepatica communis are difficult to access and cannot be easily removed. Therefore, these sites should be the targeted by postoperative radiotherapy.

**Fig 2:**
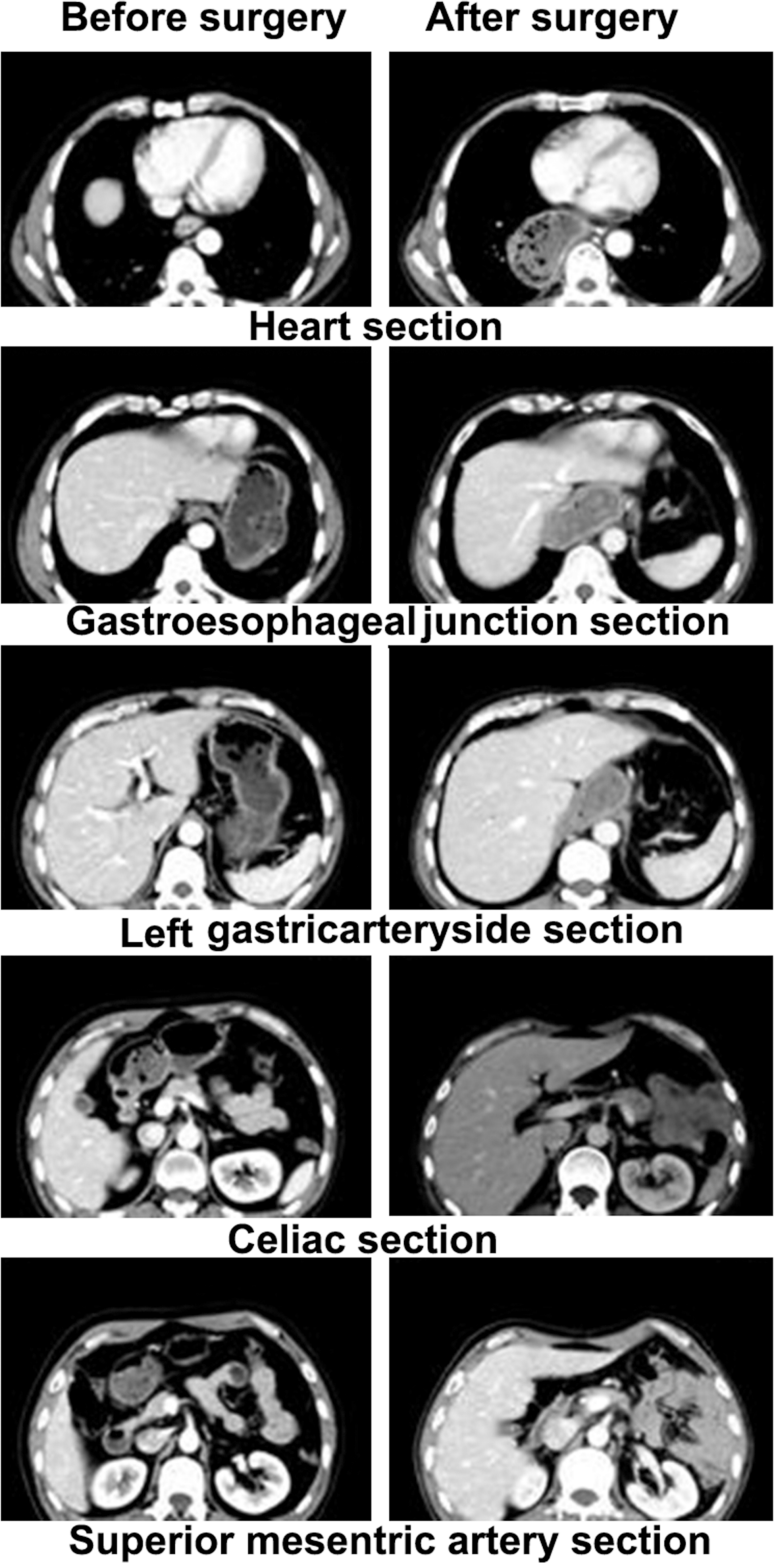
CT images of the inferior mediastinum and epigastrium before and after the radical surgery.

To date, there are no standards that define an abdominal target area for postoperative radiotherapy, but several options can be recommended. The large T target area includes the supraclavicular draining LNs, mediastinal LN, the anastomosis, and the original esophagus bed [1,6,15]; the small T target area includes the supraclavicular draining LNs, upper mediastinal draining LNs, the anastomosis, and the original esophagus bed [6]; and the conventional target area includes the esophagus bed, the subcarinal LNs, and the left gastric LNs [16]. However, regardless of the chosen target area, no significant reduction in the rates of abdominal metastasis was observed in cases subjected to postoperative radiotherapy (*P*> 0.05). Our results confirmed these previously published conclusions[1,6,15,16]. There are two possible explanations for this: first, that the draining retroperitoneal LNs were not included in the target area, and second, that the anatomical structure of the inferior mediastinum and epigastrium changed postsurgery, and thus, the corresponding LNs were obscured by gastrointestinal tissue. Fig 2 shows the anatomical structures of the inferior mediastinum and epigastrium before and after the radical surgery.

Previous studies reported improved survival rates after adjuvant radio- chemotherapy in esophageal cancer patients with more than 3 metastatic LNs [10, 17]. We showed a drastic increase in the abdominal lymphatic metastasis rates in cases with more than three affected nodes (4-fold increase) or with metastatic celiac LNs (almost 5-fold increase). Therefore, were commend adjuvant radiotherapy to the retroperitoneal draining LNs only for those cases. The lower esophageal segment could also be included when necessary in the radiotherapy target area. Conformal radiotherapy should be used to reduce the adverse gastrointestinal effects.

One of the shortcomings of this study was that the diagnosis of postoperative abdominal lymph node metastasis was dependent on regular abdominal enhanced CT, MRI, and PET-CT imaging. However, CT diagnosis of abdominal lymph nodes has an overall accuracy of 62–64% [18] and MRI has sensitivity of 71% ± 18.22, specificity of 29% ± 18.07, and accuracy of 58% ± 19.72 in this regard [19]. PET/CT for diagnosis of abdominal LN metastasis has the sensitivity, specificity, positive predictive value (PPV) and negative predictive value of FDG of 40%, 95%, 91% and 56%, respectively [20]. Most TE-SCC patients with postoperative recurrence received non-surgical treatment which limits available pathological data comparable to surgical treatment causing some deficiencies in the pathology diagnosis. In this study, strict image quality control may help to alleviate this deficiency to a certain extent. Since this study had a smaller sample size, further studies are expected to determine whether the abdominal draining LNs should be included in the postoperative radiotherapy target area. In particular, multi-center prospective large sample studies are required to compare the survival data between patients receiving preventive radiation therapy including the abdominal target area and those not containing the abdominal target area.

**Fig 3:**
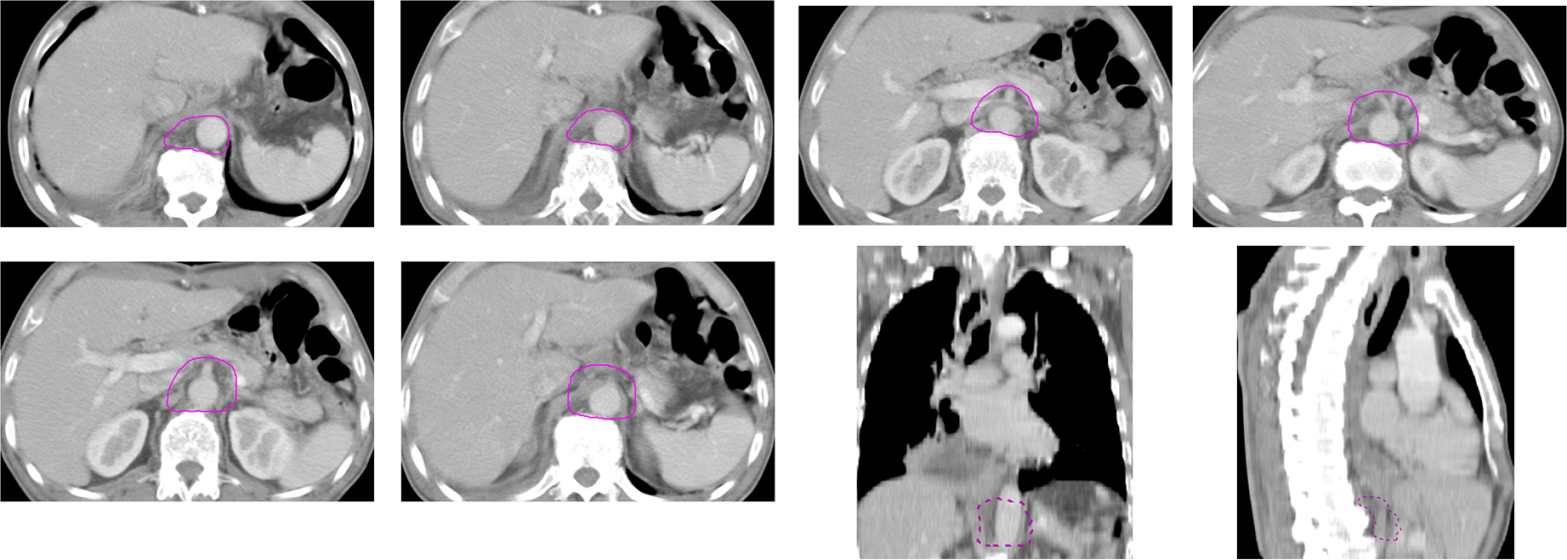
Proposed postoperative target area for radiotherapy. The lymph nodes at stations 16a1 and 16a2 of the para-aortic, truncus coeliacus, posterior surface of the pancreatic head, and arteria hepatica communis were the major sites for abdominal metastasis after radical surgery for esophageal cancers. Hence, we recommend these regions to be included in the radiotherapy target area.

We concluded that the lymph nodes at stations 16a1 and 16a2 of the para-aortic, truncus coeliacus, posterior surface of the pancreatic head, and arteria hepatica communis were the major sites for abdominal metastasis after radical surgery for esophageal cancers. Hence, we recommend these regions to be included in the radiotherapy target area (Fig 3). We also recommend that in TE-SCC cases with more than three metastatic LNs and positive for celiac lymphatic metastases, radiotherapy should be employed following the radical resection and the target area should be adjusted conformingly.

## Supporting information

S1 File Original DATA. (XLS)

S2 File Original file of approval statement. (JPG)

S3 File PLOSOne_Clinical_Studies_Checklist. (DOCX)

S4 File Translation of institutional review board statement. (DOCX)
