# Supplementary figures and images for: Patterns and rates of abdominal lymphatic metastasis following esophageal carcinoma
Source: PLoS One. 2017 Oct 10;12(10):e0185424. doi: 10.1371/journal.pone.0185424 (PMC5634562; doi:10.1371/journal.pone.0185424)

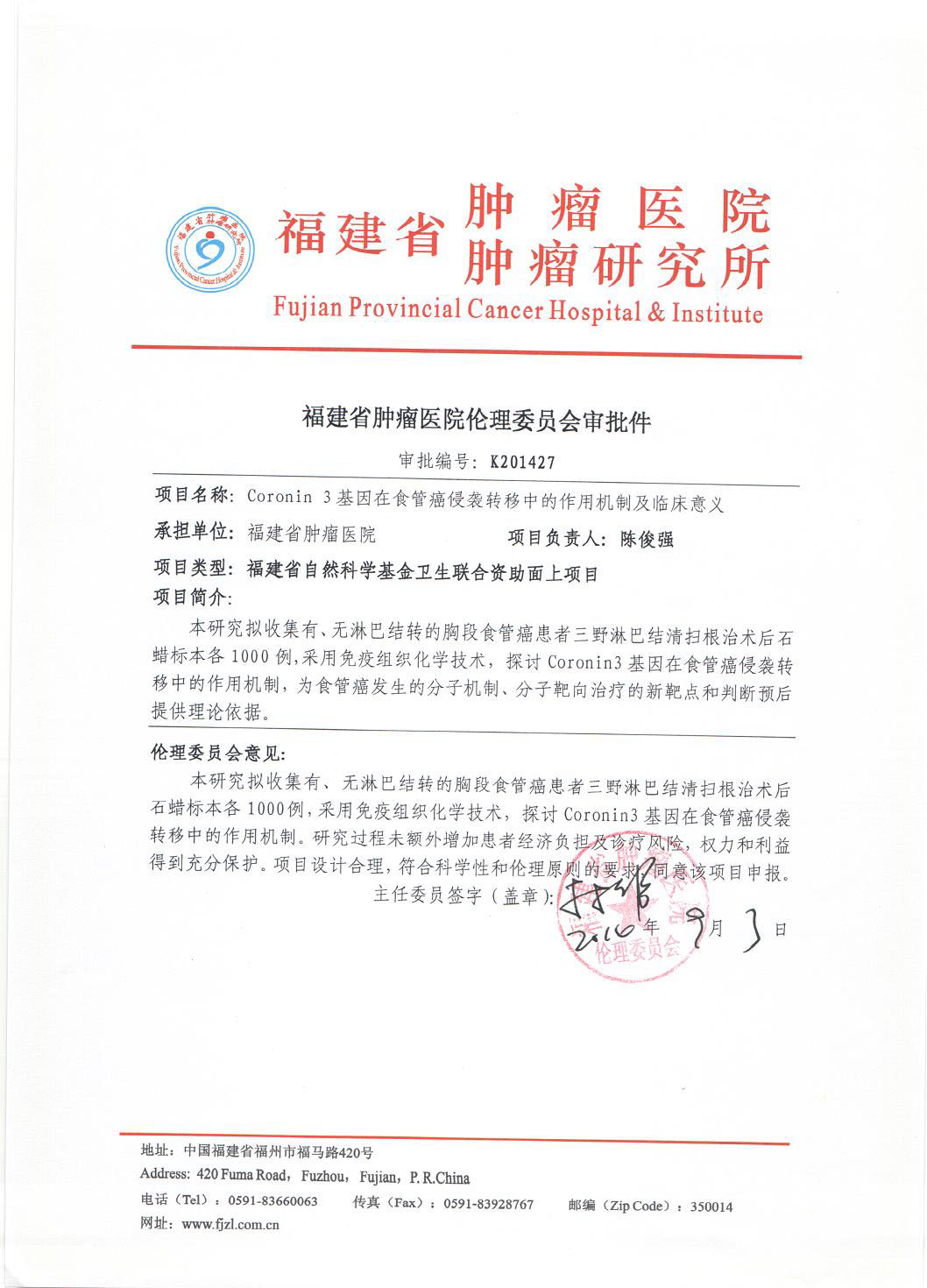

Supplement: S2 File — (JPG) [file pone.0185424.s002.jpg]
