# Supplementary material for: Patterns and rates of abdominal lymphatic metastasis following esophageal carcinoma
Source: PLoS One. 2017 Oct 10;12(10):e0185424. doi: 10.1371/journal.pone.0185424 (PMC5634562; doi:10.1371/journal.pone.0185424)
Supplement: S4 File — (DOCX) [file pone.0185424.s004.docx]

**Fujian Provincial Cancer Hospital & Institute**

**Fujian Provincial Cancer Hospital & Institute_Institutional review board approval statement: File No. K201427**

**Research title:** The mechanism and clinical value of Coronin 3 gene in the metastasis of esophageal carcinoma

**Principle Investigator:** Junqiang Chen

**Funding support:** This study is supported by grants from the Natural Science Foundation of Fujian Province (2015J01377), the Key Project of Science and Technology Foundation of Fujian Province (2011Y0014), and the Key Clinical Specialty Discipline Construction Program of Fujian, P.R.C.

**Project description:** In this study, In this study, we collected 1000 cases of paraffin specimens after three fields radical lymph node dissection in patients with thoracic esophageal cancer with or without lymph node metastasis. we investigated the effect of coronin3 gene on the invasion and metastasis of esophageal carcinoma in patients with esophageal squamous cell carcinoma by immunohistochemistry. This study will provide the theoretical basis for the molecular mechanism of esophageal cancer, the new target of molecular targeted therapy and the prognosis of esophageal cancer.

**Institutional review board statement:** This research did not increase the risk and economic burden of patients; the patients’ rights were fully protected; the project design was conducted in line with scientific and ethical principles. The institutional review board approved this project.
